# Impact of Photosensitizers Activation on Intracellular Trafficking and Viscosity

**DOI:** 10.1371/journal.pone.0084850

**Published:** 2013-12-27

**Authors:** Kelly Aubertin, Stéphanie Bonneau, Amanda K. A. Silva, Jean-Claude Bacri, François Gallet, Claire Wilhelm

**Affiliations:** 1 Laboratoire Matière et Systèmes Complexes (MSC), CNRS and Université Paris Diderot, Paris, France; 2 Laboratoire Jean Perrin-CNRS, Université Pierre et Marie Curie, Paris 6, Paris, France; US Naval Reseach Laboratory, United States of America

## Abstract

The intracellular microenvironment is essential for the efficiency of photo-induced therapies, as short-lived reactive oxygen species generated must diffuse through their intracellular surrounding medium to reach their cellular target. Here, by combining measurements of local cytoplasmic dissipation and active trafficking, we found that photosensitizers activation induced small changes in surrounding viscosity but a massive decrease in diffusion. These effects are the signature of a return to thermodynamic equilibrium of the system after photo-activation and correlated with depolymerization of the microtubule network, as shown in a reconstituted system. These mechanical measurements were performed with two intracellular photosensitizing chlorins having similar quantum yield of singlet oxygen production but different intracellular localizations (cytoplasmic for mTHPC, endosomal for TPCS2a). These two agents demonstrated different intracellular impact.

## Introduction

Photodynamic therapy uses photo-activated organic molecules (porphyrins, chlorins, phthalocyanins, etc.) with very special photo-physical properties. Owing to their triplet state, irradiation of these photosensitizers generates reactive species such as singlet oxygen [1]. The lifetime of these molecular species is very short [2,3] and their action is highly localized [4–6].

The ability of photosensitizers to target a specific cellular compartment explains their potential to modify and control cellular physiology. For example, photochemical internalization (PCI) using the photoactivation of photosensitizers that localize in endosomes, induces endosomal membrane changes, enabling release into the cytosol of an active substance stored in the endosomal compartment [7,8]. More marked changes occur if the photosensitizer is localized in the cytoplasm - particularly affecting mitochondria, endoplasmic reticulum or functional organelles - and potentially leading to cell death by necrosis or apoptosis [9,10]. This approach, called photodynamic therapy (PDT), is already used in the clinic for cancer treatment [11].

While the cellular effects of photosensitizers (cytotoxicity, membrane permeabilization, …) have been extensively explored [12,13], their impact on intracellular mechanics and trafficking are much less documented [14,15]. In particular, as the cytotoxic effect is based on the production of very short-lived reactive oxygen species, it is important to know the mechanical properties of the medium through which these species must diffuse in order to reach their target. A first approach to this issue was recently developed using a photosensitive molecule coupled to a molecular rotor whose orientation could be optically determined, allowing the diffusion properties of the molecule to be followed [15]. 

However, the relationship between diffusion and dissipation is not direct, especially in a living system [16–19]. For a Newtonian fluid at thermodynamic equilibrium, the diffusion coefficient is directly related to the viscosity, through the Stokes-Einstein relationship. This particular expression of the Fluctuation-Dissipation Theorem (FDT) can be generalized to any non-newtonain fluid, provided that it remains at thermodynamical equilibrium. However, in the intracellular medium, molecular motors regularly consume ATP, converting chemical energy into the mechanical work needed to sort and transport each cargo to its final destination [20–23]. The intracellular space is thus, by its nature, a system far from thermal equilibrium, thus challenging the validity of the fluctuation-dissipation theorem in this situation. The ratio of the chemical energy input over thermal fluctuations energy, can then be measured, as described for aging complex systems [24] as well as for the cell interior [17]. This ratio can reach 1000, implying diffusion coefficients 1000 times greater than those deduced from viscosity measurements, using FDT.

To access this ratio or, more simply, to measure dissipative and diffusive properties independently, one must be able, at the same time and in the same system, to make a passive measurement (“fluctuation”) as well as an active measurement (“dissipation”). This is the approach we used here, taking advantage of magnetic micromanipulation techniques developed in recent years to measure local viscoelasticity [25], while at the same time following the transport modalities of intracellular vesicles.

Two types of chlorins, m-THPC (hydrophobic) and TPCS2a (more hydrophilic), were chosen as photosensitizers, as their different hydrophobicities affect their intracellular localization [26–28]. Endosomes containing magnetic nanoparticles were used as both probes of the trafficking and magnetic probes for the local viscosity within irradiated cells.

Combined measurement of rheological properties and active transport, according to the photosensitizer localization and the duration of the treatment and post-treatment steps, should provide information on the intracellular mechanisms of action of photodynamic stress, a largely unexplored area.

## Results

### Simultaneous internalization of photosensitizers and magnetic nanoparticles by tumor cells

**Figure 1:**
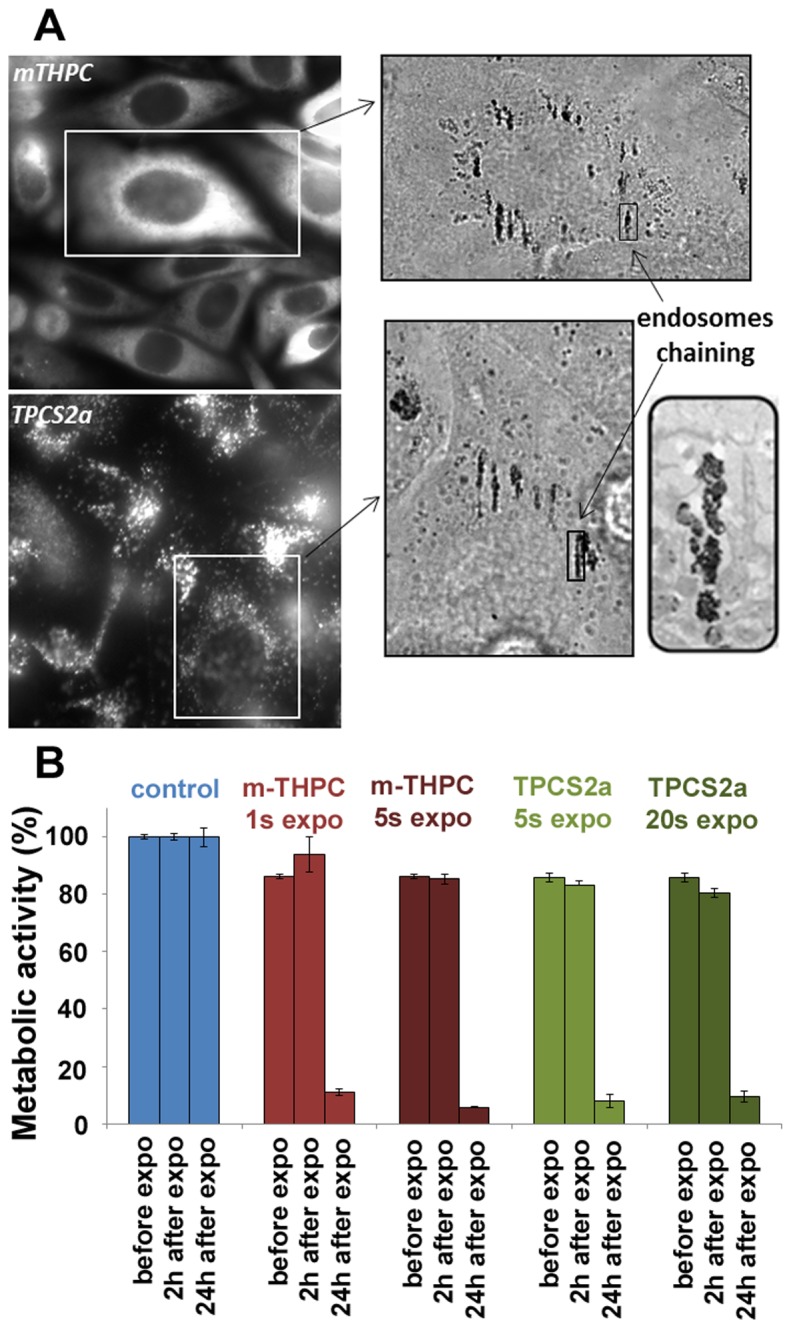
Intracellular localization of the photosensitizers and the magnetic nanoparticles and metabolic activity. (A) Co-internalization, in PC3 tumor cells, of photosensitizer molecules (mTHPC and TPCS2a) and magnetic nanoparticles. mTHPC localizes in the cytoplasm while TPCS2a is found in vesicular structures. The magnetic nanoparticles are concentrated in late endosomes and lysosomes, and align in the direction of the applied magnetic field, as shown by electron microscopy (inset). (B) Metabolic activity was not modified 2 hours after exposure, regardless of the conditions. The subsequent steps therefore involved viable cells. However, a noteworthy cytotoxic effect was detected the following day, with more than a 80% fall in cellular activity. The error bars represent the standard error of the mean (SEM).

Incubation of cells with magnetic nanoparticles and photosensitizers led to internalization of both components (Figure 1A). Each cell contained an average of 10^7^ nanoparticles, equivalent to 10 pg of iron. The nanoparticles penetrate through the endocytic pathway and cluster within endosomes, as previously demonstrated [29–31].

Then, if a homogeneous magnetic field is applied, each endosome acquires a magnetic moment which aligns with its neighbors under the effect of magnetic dipole forces. This creates small intracellular chains that can be manipulated via the external magnetic field. The two photosensitizers are also internalized, at dose equivalent of 0.2 fmoles per cell on average. However, m-THPC spreads in the cytoplasmic compartment, while TPCS2a is found in the endosomal compartments.

After irradiation (deposited energy between 1.5 and 30 J/cm^2^), the cells’ metabolic activity was not reduced during the first two hours (Figure 1B). In contrast, a strong decrease in cell viability was detected 24 hours after exposure.

We thus obtained magnetic sensors inside cells containing photosensitizers with different intracellular targets, within a range of concentrations at which treatment is cytotoxic at long times.

### Probing intracellular trafficking with magnetic endosomes : Diffusion measurements

**Figure 2:**
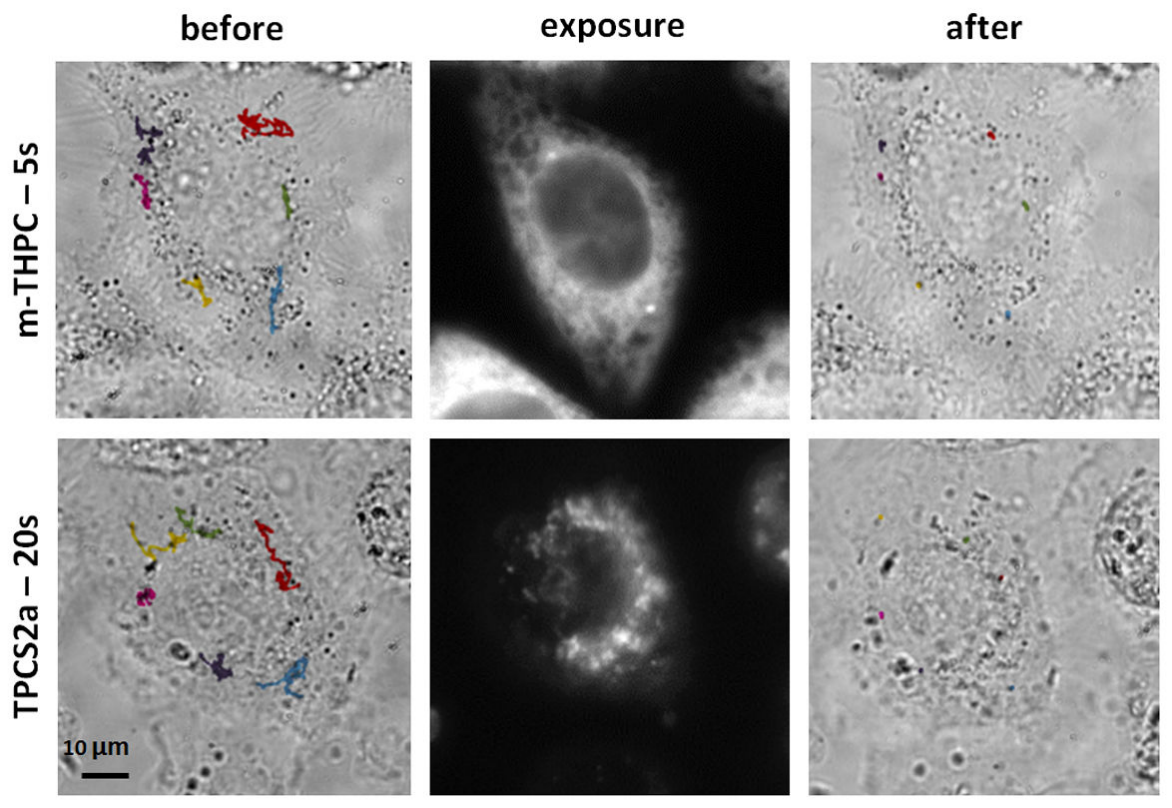
Monitoring of intracellular trafficking. The dark compartments (endosomes filled with magnetic nanoparticles) were followed for 100 s and their trajectories (before and after exposure) were superimposed on the image of the cell obtained at the outset of the monitoring phase. Movements were clearly inhibited by the action of the photosensitizer (mTHPC and TPCS2a, 5 s and 20 s of exposure, respectively).

We first aimed at exploring the impact of photodynamic stress on intracellular trafficking. Endosomes loaded with magnetic nanoparticles are easily detectable by optical microscopy. It is possible to track their position (*x*,*y*)  in a time-resolved manner (image capture every 100 ms for 100 s). In figure 2, left images show a few example of endosome trajectories (color lines) superimposed to cells before photo-activation. After photo-activation, one can see a dramatic decrease in trafficking (strong shortening of all track lengths).

To quantify such an effect, the mean square displacement <*Δr*²(*t*)>  was calculated for each trajectory (see Methods, equation 4).

**Figure 3:**
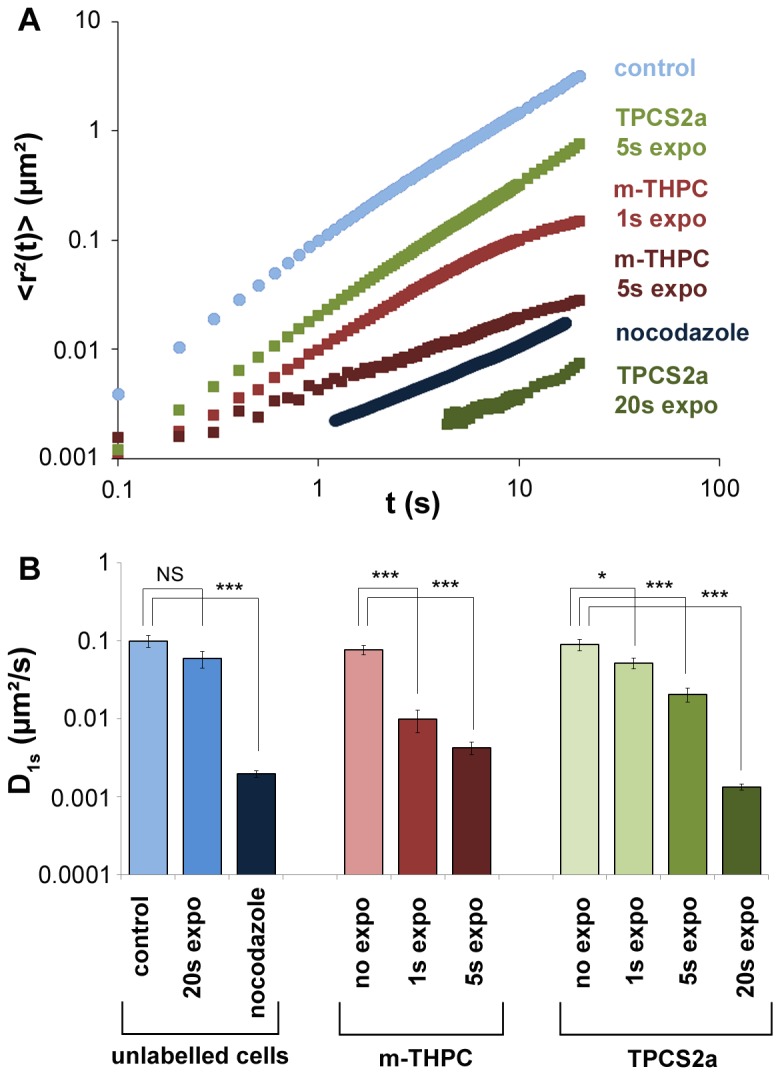
Quantification of intracellular trafficking. (A). The mean square displacement (<*Δr*²(*t*)> ) was calculated for all conditions according to equation (4) and averaged over 30 independent measurements. For means of clarity, only the main conditions are represented: unlabeled cells non exposed (control, light blue), unlabeled cells treated with nocodazole (dark blue), cells treated with m-THPC and exposed for 1s (red) or 5s (dark red), and cells treated with TPCS2a and exposed for 5s (green) or 20s (dark green). (B). Diffusion coefficient (*D*_1s_) defined in equation (1). It corresponds to the value of the mean square displacement at 1s (<*Δr*²(1s)> ). It is shown for all tested conditions, the ones presented in part A, as well as additional ones, including unlabeled cells exposed for 20s (blue), cells treated with both photosensitizers (m-THPC, light red and TPCS2a, very light green) but non exposed, and cells treated with TPCS2a and exposed 1s (light green). The error bars represent the standard error of the mean (SEM), NS (Non Significant) corresponds to p>0.05, * corresponds to 0.01<p<0.05, and *** corresponds to p< 0.001.

Figure 3A shows the <*Δr*²(*t*)>  for different treatment conditions : control cells and microtubule disrupted cells, m-THPC treatment, TPCS2a treatment, both at various exposure times. In all cases <*Δr*²(*t*)>  obeys a power law with time:

$$<\Delta r²(t)>\;=2D_{1\text{s}}t^{\alpha }$$ (1)

α characterizes the type of movement (confined*α*<1, diffusive*α*=1, directed*α*>1; more details are given in the Methods section); *D*_1s_reflects the amplitude of motion at the characteristic time t=1s. Figure 3B summarizes all mean *D*_1s_ values extracted from the mean square displacements data presented in Figure 3A, and includes all complementary controls. *D*_1s_fell when the microtubules were depolymerized and when the photosensitizers were irradiated, the fall being proportional to the dose of irradiation, and demonstrating a reduction of intracellular motions. The value of the exponent *α* (extracted from the fitting of the curves shown in Figure 3A) provides information related to the mode of the observed motions. In untreated cells, exponent *α*≈1.3 is a signature of a super-diffusive motion related to active transport of the endosomes along the microtubule network by its associated molecular motors [16,32–34]. Indeed, the same cells treated with nocodazole, a microtubule depolymerizing agent, exhibit an average exponent of 0.8, indicating confined movement. The same effect was observed after irradiation, increasing with the amount of light energy deposited. With m-THPC, irradiation for 1 s or 5 s reduced the exponent to 1.0 or 0.6, respectively, while with TPCS2a irradiation for 1s, 5 s or 20 s reduced the exponent to 1.2, 1.1 or 0.7, respectively. 

**Figure 4:**
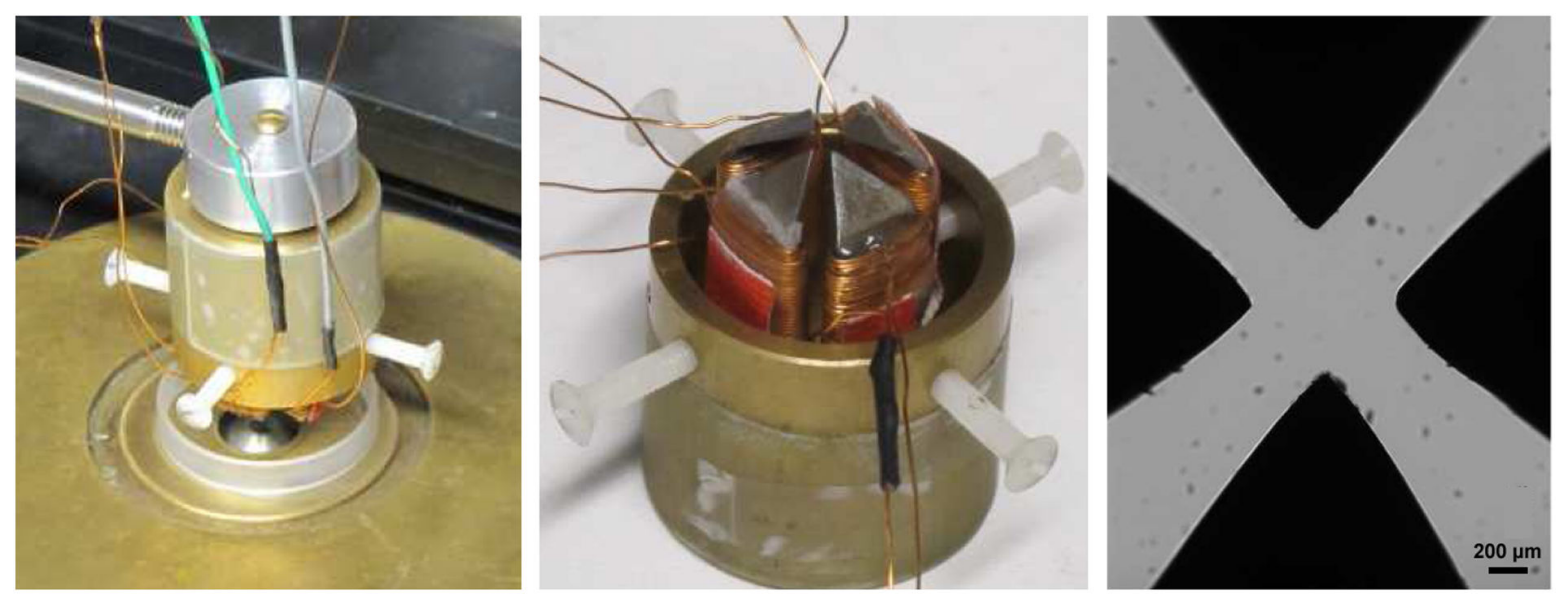
Magnetic device. (Left): The magnetic set-up was adapted to an inverted microscope with a plan 63x oil immersion lens. (Middle): It consists of two pairs of home-made coils magnetizing four engineered soft iron pieces. (Right): The space between the magnetic pieces is 0.6 mm, creating a strong magnetic field in the center (up to 70 mT for the maximum applied current of 1A).

At this stage of our understanding, the drop in endosomes trafficking after irradiation could be due either to an inhibition of the motor-mediated out-of-equilibrium endosomes motions, or to a stiffening of the mechanical environment, causing an increase of the local viscosity, as previously reported [15]. In this latter case, the viscosity η, should rise by the same factor as the recorded drop in displacements (that is up to a hundred-fold), as further discussed in the Discussion section. The next step therefore requires performing dissipative measurements of the endosomes surrounding viscosity to validate one or the other hypothesis.

### Probing the intracellular viscosity with magnetic manipulation

**Figure 5:**
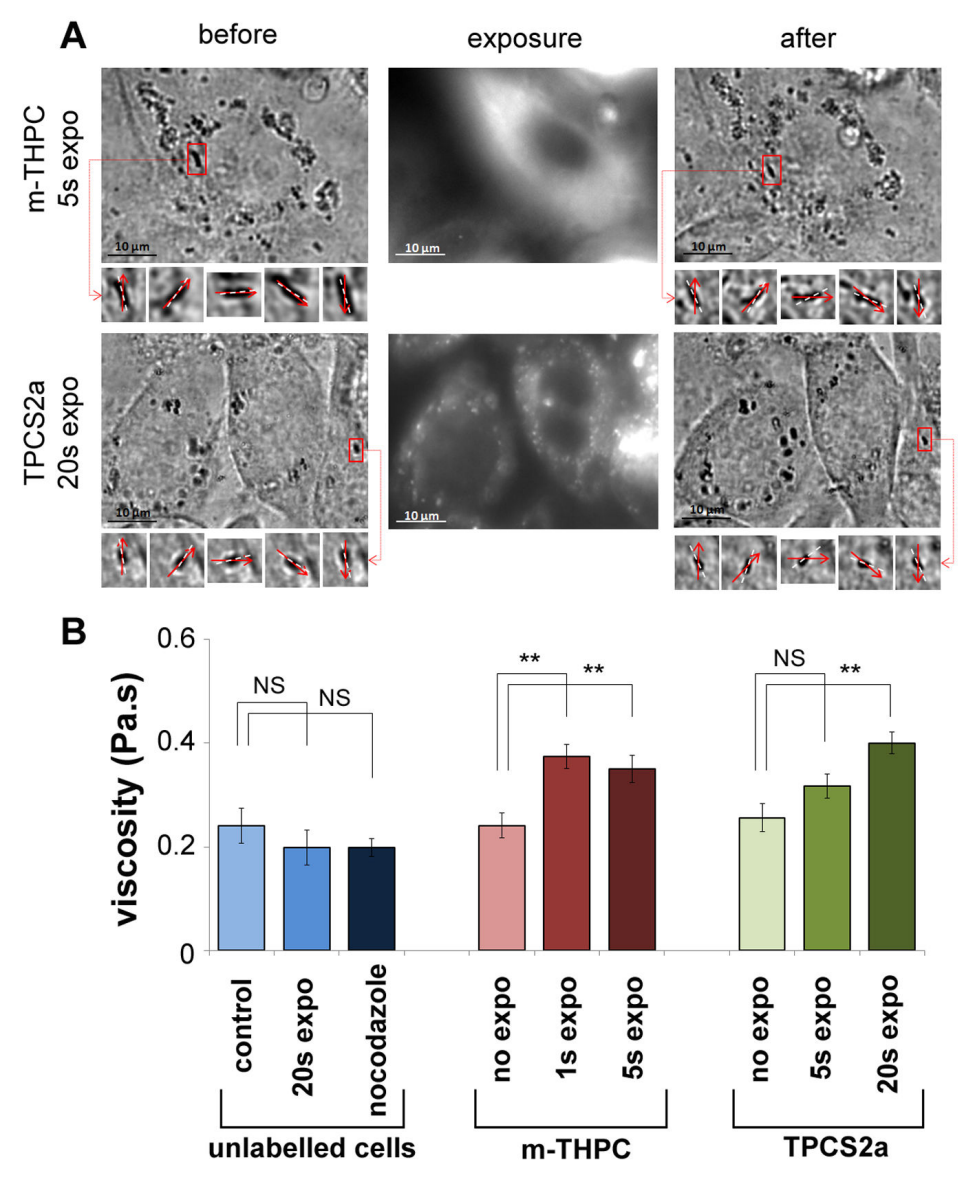
Measuring intracellular viscosity. (A) Example of rotations of a chain of magnetic endosomes before and after exposure, for the mTHPC (top) or the TPCS2a (bottom) photosensitizers. The phase lag φ between the direction of the magnetic field (dotted white line) and the direction of the selected chain (red arrow) are indicated in each case. For these two examples, the viscosity around the observed chain slightly increased after treatment, from 0.11±0.01 Pa.sto0.18±0.03 Pa.s Pa.s in the mTHPC case, and from 0.25±0.05 Pa.sto 0.55±0.04 Pa.sin the TPCS2a case. (B) Mean viscosity (averaged over 25 independent measurements at least) is shown for each condition (for unlabeled control cells, for unlabeled cells exposed for 20s, for unlabeled cells treated with nocodazole (10µM, 30min), for cells treated with m-THPC (without exposition, or exposed for 1s and 5s), for cells treated with TPCS2a (without exposition, or exposed for 1s, 5s, and 20s). Controls and cells treated with photosensitizers but not exposed all display the same viscosity mean value, in order 0.25 Pa.s. After nocodazole treatment, the mean viscosity value slightly decreases to 0.2 Pa.s but not significantly, while after exposure of the photosensitizers, it increases to 0.4 Pa.s at maximum. This increase is significant considering a confidence level of p<0.01. The error bars represent the standard error of the mean (SEM), NS (Non Significant) corresponds to p > 0.05, and ** corresponds to p<0.01.

Such a measurement of the viscosity requires an externally applied stress on an internal probe. The idea here is to exploit the magnetic properties of the labeled endosomes to make them align in chains and impose the chain rotation by an external magnetic rotating field: the lag in time for the endosomal chains to align with the rotated field is a measurement of viscosity. The technical difficulty however is to produce a rotating magnetic field strong enough to align magnetic endosomes, and deliver a sufficient magnetic torque to permanently rotate these chains. To do so, we designed a miniaturized magnetic device (Figure 4), consisting of a set of 4 home-made small coils magnetizing 4 soft iron engineered pieces separated by only 0.6 mm. All details and pictures are presented in the Methods section “magnetic device”. Thanks to the small spacing, the magnetic field created in the center can be tuned between 0 to 70 mT and its direction can be adjusted within the cell sample plane, by tuning the current supplying each coil. In particular, if the two pairs of coils are supplied with an alternating current 90° out of phase, the produced magnetic field rotates in the cells plane. Chains of endosomes then undergo a magnetic torque (defined in the Methods) which forces them to rotate along with the field (if the latter is strong enough), with an angular delay *φ* (between the direction of the chain and the magnetic field, examples given in Figure 5A). This delay is due to the opposition to rotation of the surrounding medium (creating a viscous restoring torque proportional to the viscosity*η*, details also given in the Methods). The measure of *φ* thus provides direct access to the viscosity *η* according to

**Figure 6:**
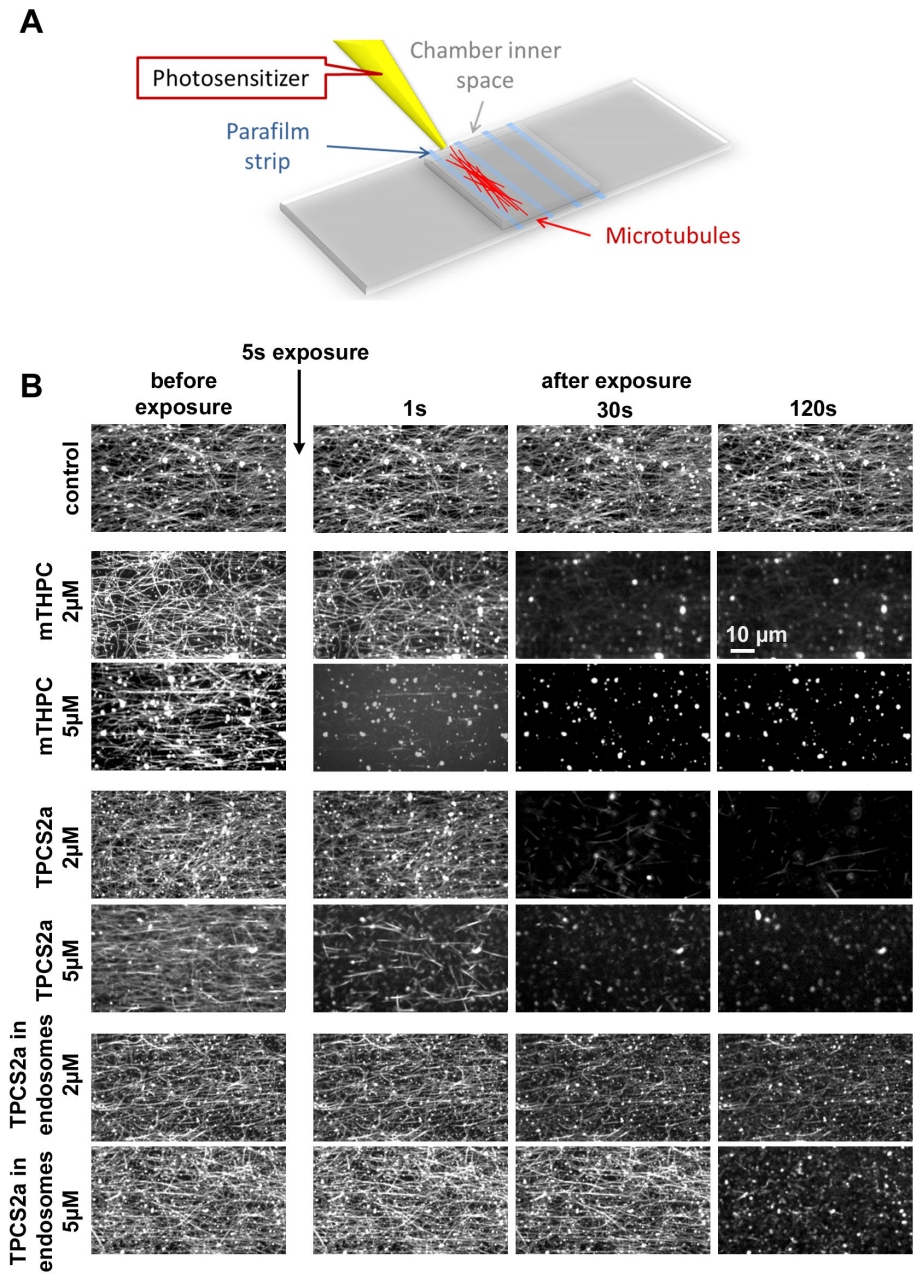
Monitoring microtubule depolymerization. (A) Schematic representation of the cover glass chamber used in the *in* *vitro* microtubule assay featuring an inner space filled by capillarity. (B) Network of reconstituted microtubules immersed in a bath containing a photosensitizer. With m-THPC or TPCS2a at a concentration of 2 or 5 µM in solution, complete depolymerization occurred very rapidly, within less than 10 seconds. We can see first the destabilization (1s) of the microtubule structure and second the depolymerization. When TPCS2a-labelled endosomes are used as photodynamic agent, at the same concentration, the depolymerization is strongly delayed.

$$\eta =\frac{\mu_{0}m^{2}}{32\pi \kappa }\frac{\sin\left(2\varphi \right)}{F}$$ (2)

where*μ*_0_=4π*10^−7^kg m A^−2^s^−2^, m is the endosomes magnetic moment at the applied field, *F*the frequency of the field, and κ a previously calibrated geometric factor (*κ*=6.7, 14.9 and 24.7 for chains containing 2, 3 and 4 endosomes, respectively [35,36]. 

To retrieve quantitative viscosity values, we also needed an independent measurement of the magnetic moment of each endosomes. To do so, we extracted them from the cell and injected them into a magnetophoresis chamber subjected to a calibrated magnetic gradient (280 T/m). Each endosome migrated with an average speed of about 30 µm/s, which corresponds to a magnetic moment for an applied field of 50 mT (the one selected for the experiments) of*m*=(1.8±0.2)*10^−16^A m^2^.

Before photoactivation, the mean value of the viscosity retrieved was 0.25±0.03 Pa.s. The error corresponds to the standard error of the mean (SEM), averaged over more than 25 independent measurements. One must note however that the standard deviation is large (in order 45%), due to the variability of the viscosity within the cytoplasm.

Neither the presence of a photosensitizer without irradiation, nor cell irradiation without a photosensitizer, affected this value. After cell exposition to the photosensitizer and irradiation, the intracellular viscosity slightly increased to values in order 0.4 Pa.s (all values are shown in Figure 5B), and the increase was significant considering a confidence level ofp<0.01. By contrast, after nocodazole treatment, the mean viscosity value slightly decreases to 0.2 Pa.s but not significantly.

Therefore, while the diffusion coefficients D_1s_ decreased after exposure by a factor at least 20, and up to 100, with a significance at a confidence level of p<0.001 for all measures, the viscosity values for irradiated cells only increased by a factor 2 or lower, compared to the ones of untreated cells, and with less significance (p<0.01). This means that the irradiation strongly impact the endosomes transport but not their surrounding viscosity, bringing the system back to a situation close to equilibrium. These results could be explained by the inhibition of molecular motors mediated transport on microtubules, and we therefore investigated further the impact on the microtubules of photoactivation with the investigated photosensitizers. 

### Microtubules: targets of the photodynamic effect

Microtubules are in perpetual remodeling [37,38] and have yet been identified as a target during photodynamic treatment [39–41] and, at this stage, it is logical to hypothesize that an action of the two photosensitizers on the microtubules network may be responsible for the observed decrease in active trafficking. As the reactive species photo-induced via the photosensitizers present a very short action range, their subcellular localization may strongly impact their respective effects. For TPCS2a, unlike for m-THPC, the produced oxygen species must first cross the membrane of the endosome before coming close to the microtubule. However, their ability to react with these membranes is well-known - and used as basis of PCI [42,43]. Consequently, it is expected that the diffuse-labelled photosensitizer m-THPC reacts faster with the microtubules. To test this possibility, we developed an *in vitro* system of reconstituted microtubules and followed their dynamics after irradiation. Within the range of intracellular photosensitizer concentrations (0.2 fmoles per cell), irradiation had a significant depolymerizing effect (Figure 6): immediately after 5 s of irradiation, with a m-THPC concentration of 2 or 5 µM, the microtubule depolymerization took only a few seconds. For TPCS2a, two kinds of experiments were performed, with the aim to mimic, in a reconstructed system, the effects observed within cells. First, results similar to those of m-THPC were obtained with TPCS2a in solution within the same concentration range. This highlights that both photosensitizers, which have similar singlet oxygen quantum yields, act similarly in solution as photo-depolymerization agent. However, within cells, the subcellular localization of TPCS2a is strongly different from that of m-THPC : it remains sequestrated within endosomes. Then, in a second experiment, TPCS2a-labelled endosomes were prepared and used as photo-depolymerization agent in the same way as free TPCS2a (i.e. within the same photosensitizer concentrations, determined by fluorescence spectroscopy). The TPCS2a activity is then strongly slackened as compared to free TPCS2a, corroborating the trapping role of the endosomal membrane for the photo-induced reactive species. 

## Discussion

Here we examined the intracellular impacts of the photodynamic effect. Photosensitizers are used in various biomedical applications, from induction of cell death for cancer therapy (PDT) to triggering the release of endocytised macromolecules (PCI) [9,44]. We chose two “standard” photosensitizers, mTHPC and TPCS2a, for this study. These two molecules, which have very similar chemical formulas (chlorin rings), differ only by a terminal functional group. This simple difference makes TPCS2a less hydrophobic than mTHPC and strongly modifies the intracellular localization (cytoplasmic for mTHPC and endosomal for TPCS2a). It is interesting to note that the therapeutic functions of the two photosensitizers also differ: m-THPC (FOSCAN) is used in the clinic, providing effective PDT, while TPCS2a is used within the context of drug delivery as PCI agent, to induce endosomal membrane rupture and the release of active molecules into the cytoplasm. We used this well-controlled model to study the effect of photodynamic stress on intracellular mechanics.

Viscosity changes of the intracellular medium following irradiation have only been studied previously in a single model system using spectrally resolved fluorescence measurements of a porphyrin-dimer-based molecular rotor [15]. Such an approach is only valid if the measurement is made at thermodynamic equilibrium. This does not hold if the probe is localized in endosomes, which appears to be the case in this latter model: the rotor-photosensitizer appears as a small dot in the cytoplasm, suggesting that it is localized in vesicles (presumably endosomes). The observed diffusion of the molecule thus reflects both its diffusion within the vesicle and the diffusion/transport of the vesicle itself.

Here, we chose to investigate the properties of the intracellular microenvironment after photosensitizers activation by using a dual approach: on the one hand, we measured viscosity directly from the angular delay between a chain of magnetic spheres (endosomes) and a rotating magnetic field, and, on the other hand, we followed the spontaneous movement of the same objects and thus derived a diffusion coefficient. 

For a Newtonian fluid at thermodynamic equilibrium, the diffusion coefficient D is related to the viscosity η of the surrounding medium according to the Stokes-Einstein equation:

D=kT/6πηa (3)

where *a* is the endosome probe diameter. Note that *D* corresponds to our parameter *D*_1s_in the case of Brownian diffusion where exponent α is equal to 1, and equation (1) becomes the equation of diffusion<*Δr*²(*t*)> =2*Dt*. In our present case, the intracellular medium surrounding the endosomes is not a Newtonian fluid (because of the cytoskeleton structure and the presence of membrane compartments) and the coefficient *D*_1s_ only represents the amplitude of diffusion at the characteristic time t=1s. Also, strictly speaking, the viscosity of the medium cannot be defined since the relationship between velocity and drag force is not linear and η must be seen as a viscous coefficient at a characteristic time. Nevertheless, at thermal equilibrium, a rough generalization of the Stokes-Einstein relation may be written as equation (3). Let us then derive the viscosity from the diffusive measurements according to Stokes Einstein equation, for the purpose of demonstration: before treatment, one retrieves*η*_from diffusion_=0.007 Pa.s, and after treatments, for the different conditions, *η*_from diffusion_=0.07 Pa.s(mTHPC 1s), *η*_from diffusion_=0.2 Pa.s(mTHPC 5s), *η*_from diffusion_=0.03 Pa.s(TPCS2a 5s), *η*_from diffusion_=0.5 Pa.s(TPCS2a 20s). The effective viscosity deduced from the diffusivity, increase after irradiation. However, as the system is out equilibrium it does not reflect a real increase in the viscosity, it corresponds instead to a decrease of the intracellular trafficking. The value for untreated cells is far removed from the one obtained with the direct dissipation measurements*η*=0.25 Pa.s), while after exposure, the derived values get closer the measured ones (*η*=0.4 Pa.s). Equation (3) is therefore strongly violated in untreated cells, indicating a deviation from equilibrium linked to ATP-dependent phenomena such as directed trafficking mediated by molecular motors. After irradiation, the intracellular environment gets closer to equilibrium (in particular for the longest exposure with TPCS2a). This provides indirect evidence that photosensitizers activation inhibits intracellular trafficking, bringing the system closer to thermodynamic equilibrium, where diffusion is controlled primarily by thermal energy. Finally, it must be noted that this study demonstrates that deriving a viscosity from diffusive measurements is not valid, and that any conclusion concerning an increase in viscosity after photosensitizers activation measured with such passive approach [15] must be considered with care. It should also be noted that the measured viscosity here increased only slightly, by a factor less than 2, but significantly (due to the important number of measures performed, always more than 25). 

It is interesting to compare the magnitude of the effects between the two photosensitizers for the same dose of irradiation. The two photosensitizers have the same quantum yield of singlet oxygen, so that any differences between them can only result from differences in their modes of interaction with the cell. Thus, 5s of irradiation of cells loaded with TPCS2a had little effect on peri-endosomal viscosity or endosome diffusion, while the same irradiation of cells loaded with m-THPC strongly modified both parameters. These results were expected, given the cytoplasmic localization of m-THPC, as its immersion in the microtubule network makes it easier for short-lived singlet oxygen molecules to reach their target. In contrast, the singlet oxygen produced by TPCS2a, being located within endosomes, must diffuse through the endosome membrane prior to hit a cytoplasmic target such as microtubules.

To demonstrate further this hypothesis and to give direct evidence that the photosensitizer excitation impacts the microtubules network, we directly tested the irradiation on a reconstituted network of microtubules immersed in a bath containing the two photosensitizers in solution at concentrations equivalent to those measured inside the cell. The effect was immediate in the whole concentration range: irradiation entirely depolymerized the microtubules within a few seconds. At the lower concentration range, depolymerization occurred more slowly and was sometimes only partial, but the microtubule structure was nevertherless strongly modified. This observation, showing that microtubules are targets of photodynamic therapy, supports the results of some previous studies. Indeed, microtubule network has been identified as a prime target for anticancer photodynamic therapy [11], so that microtubule inhibitors have been tested as enhancers for photodynamic therapy [45]. Interestingly, these studies, corresponding to the early stages of the process of PCI invention, lead the authors to consider lysosomes and microtubules as main targets. In fact, the localization of the photosensitizer dictates which of these entities the preferred target is. 

Anyway, the inhibition of the microtubules polymerization within NHIK 3025 cells during photodynamic treatments has been studied with various photosensitizers, in particular with TPPSn [39,40]. Actually, cytoskeleton damages, notably on microtubules network, during PDT are partly involved in the initiation of the cell death [41,46]. More recently, the binding of porphyrin derivative photosensitizers to tubulin dimers was described [47] together with the photo-induced modification of the physical properties of the MTs network by reactive species generated directly by the light interaction with the fluorophore labeling the tubulin [48]. 

Finally, we can support that, regarding to their subcellular localization, the photodynamic effect of m-THPC and TPCS2a can be deciphered by two different scenarios. MTs are primary targets for the cytosolic photosensitizer m-THPC, whereas an important quenching of this effect results from the entrapping of the TPCS2a within the endosomal membrane, which constitutes a competitive target. As a consequence, larger doses of photosensitizer or light are necessary to overcome this barrier for endosomal TPCS2a to approach the potency of mTHPC's ability to disrupt microtubules and cause drastic decreases of active trafficking

## Conclusion

Here the dual effects of photosensitizers activation on the viscous and diffusive intracellular properties are evidenced for the first time.

The dominant effect concerns the inhibition of the endosomal trafficking, which is found up to 100-fold lower after irradiation. By contrast, the viscosity did not increase enough to explain such an effect, demonstrating that the ligand (or binding site) for the photosensitizer is the major determinant of its ability to disrupt intracellular transport through the depolymerization of microtubules.

Also interesting is the combining of active and passive measurements which allow us to compare the viscosity extrapolated from the passive diffusive measurements (assuming that the system is at thermal equilibrium) and the measured one: they differ by two orders of magnitude in control cells, which are clearly out-of-equilibrium systems; by contrast, they come closer to each other after irradiation: thermal equilibrium is approached, through the partial inhibition of the active motions mediated by molecular motors.

Finally, at the same concentration, cells treated with m-THPC are more impacted that the ones treated with TPCS2a, in keeping with their intracellular location and their use for cell treatments. 

## Materials and Methods

### Photosensitizers

TPCS2a (Benzenesulfonic acid, 4,4'-(7,8-dihydro-15,20-diphenyl-21H,23H-porphine-5,10-diyl)bis-) was kindly provided by PCI Biotech AS, Oslo, Norway, as a powder with a purity of more than 98%. m-THPC (5,10,15,20-tetra(3-hydroxyphenyl)chlorin), was purchased from Frontier Scientific (Logan UT, USA). Photosensitizer stock solutions were prepared in ethanol. All the solutions were handled in the dark.

### Cell labeling

The cells are Human Prostatic Cancer Cell lines (PC-3, ATCC® CRL-1435™) and were cultured in T75 flasks at 37°C in 5% CO_2_ in Dulbecco’s modified Eagle’s medium (DMEM) completed with 10% fetal bovine serum and 1% penicillin and streptomycin antibiotics.

Tumor cell were brought to confluence in glass-bottomed 35-mm Petri dishes for high magnification immersion microscopy (63X objective). The cells were then incubated with Roswell Park Memorial Institute medium (RPMI) containing citrate coated magnetic nanoparticles (maghemite, negatively charged, 8 nm in diameter, iron concentration 2mM)[31,49] and the photosensitizers (m-THPC or TPCS2a) at a concentration of 5µM. After incubation for 2 hours, the cells were rinsed three times and placed in complete DMEM. The next day they were irradiated and observed under a thermalized inverted microscope (at magnification 63x).

To assess the influence of the microtubules on the intracellular trafficking and viscosity, cells were treated with a microtubule-disrupting drug, namely nocodazole (Sigma Aldrich, 30 min, 10 mM).

### Irradiation

The cells were exposed to light under the microscope with a wavelength of 470 nm, corresponding to one of the two excitation peaks of the photosensitizers used. As the photosensitizers then emit at 650 nm, a fluorescence image is recorded systematically in order to observe the intracellular localization and intensity of the photosensitizers. Irradiation times of 1s, 5s and/or 20s were used, corresponding to deposited energies of 1.5 J/cm^2^, 7.5 J/cm^2^ and 30 J/cm^2^. The fluorescent pictures were taken with a camera Cool Snap (CoolSnap HQ). The effect of irradiation on cellular metabolism was studied by using the Alamar blue test for metabolic activity.

### Monitoring of intracellular trafficking

We videomonitored the cells at a acquisition rate of 10fps during 100s, with a high speed camera Phantom (Phantom V9.1 Vision Research), to observe the spontaneous motion of the endosomes. Their trajectories were extracted using Image J software (Analyse Particles module), and the coordinates (*x*(*t*),*y*(*t*)) retreived. The mean square displacement <*Δr*²(*t*)>  of each trajectory was then computed with Excel software, according to : 

$$<\Delta r²\left(t\right)>={\langle {\left(x\left(t+t'\right)-x\left(t'\right)\right)}^{2}+{\left(y\left(t+t'\right)-y\left(t'\right)\right)}^{2}\rangle }_{t'}=\langle \Delta x_{}^{2}\left(t\right)\rangle +\langle \Delta y_{}^{2}\left(t\right)\rangle$$ (4)

where brackets denotes averaging.

In most situations, <*Δr*²(*t*)> follows a power law with time, <*Δr*²(*t*)> =2*D*_1s_ *t* ^*α*^, defined in the Results as equation (1). Generally speaking, the value of the exponent α characterizes the diffusive behavior in the cell: *α*=1corresponds to a pure diffusive behavior in a newtonian fluid at thermal equilibrium; the motion is sub-diffusive (confined) for an exponent*α*<1, and super-diffusive (directed) for an exponent 1<*α*<2 (*α*=2corresponding to ballistic motion) [50].

### Extraction of endosomes and determination of the magnetic moment by magnetophoresis

The day after incubation, cells were detached with trypsin and centrifuged at 4°C for 5min (250 g). The cells were resuspended in HB (250mM sucrose, 3mM imidazole) plus 1mM DTT and 1/1000 PIC (protease inhibitor cocktail) and centrifuged twice. After the last centrifugation, the cells were resuspended in 1 mL of buffer and disrupted by extruding 10 times through a 23G needle, a treatment that leaves nuclei intact. Then this cell lysate were centrifuged 6 min at 700 g (4°C) to remove the nuclei. The post-nuclear supernatant were then placed against a magnet for 1h and the magnetic endosomes were collected in BRB-taxol-DTT (1X-10µM-5mM).

To measure the magnetization of the endosomes, a thin rod of nickel (50µm) was trapped in a chamber made of two cover glasses, creating a 150mT magnetic field and 280 mT/mm magnetic gradient (characterized with magnetic beads of known magnetization) in the window of observation, 50µm apart from the rod. A small volume (10µl) of the solution containing the magnetic endosomes was inserted in the chamber and the rod was magnetized by an external magnet. The velocity of the endosomes was measured, and the magnetization of the endosomes deduced by equilibrating the magnetic force with the viscous drag (see 51 for more details). The magnetic moment of endosomes at saturation was found equal to *m*_*sat*_=(3.3±0.5)*10^−15^A m^2^(equivalent to (26±4)*10^3^ nanoparticles per endosome). This saturation is reached for applied magnetic field over 200 mT. At 50 mT for instance (the field selected for the experiments here), the nanoparticles are magnetized at only 55% of their saturation value (see for instance [REF] for a magnetisation curve) and the magnetic moment per endosome equals*m*=0.55*m*_*sat*_=(1.8±0.2)*10^−15^A m^2^.

### Magnetic endosomes chaining

In the presence of a magnetic field, the magnetic endosomes should align in the direction of the field to form small chains within the cytoplasm. Indeed, the interaction energy between two endosomes,$E_{dipole-dipole}=-\frac{2\mu_{o}m^{2}}{a^{3}}$ , where *a* is the endosome diameter (0.6 µm), is in order10^3^ *k*_*B*_ *T*: once formed inside the cell, the chains are stable against thermal fluctuations. In a 50 mT magnetic field, chains from two to four endosomes were observed within the cells. 

### Magnetic device

The magnetic device to apply a rotational magnetic field was specially designed in the laboratory and is composed of four coils which cores are made of soft iron. Pictures of the devices are shown in the Figure 4. The coils are connected by pairs and supply by an alternative current. The space between each tip is about 600 microns yielding a strong magnetic field (50 mT for the one selected in this study, corresponding to a 0.65 A current; possibly reaching 70 mT for a 1A current) in the plane of the cells. The magnetic field $\vec{B}$ rotates if the two pairs of coils are supplied with sinusoidal currents displaying the same frequency but 90° out of phase : the generated magnetic fields in the *x*−*y*plane, *B*_*x*_and *B*_*y*_ are sinusoidal with the same frequency, but with a phase lag of 90°, meaning that *B*_*x*_=0 when *B*_*y*_ is maximum, and reciprocally. The resulting magnetic field thus display always the same modulus, but its direction rotates in the *x*−*y* at the frequency of the currents applied. A light-emitting diode (LED) is mounted at the top of the device to illuminate the sample. This same diode is connected to the input signal, to trigger its turning of at the exact instant when *B*_*x*_=0 (and thus calibrate the magnetic field angle for all the captured frames). Finally, the whole set-up was adapted to a micromanipulator mounted on a Leica DMIRB microscope (thermostated at 37°C by cube&box, Life Imaging Services), and was systematically sterilized prior to use. 

### Magnetic rotation of the chains : principles and viscosity measurement

When the magnetic field rotates, the applied magnetic torque depends on the angle φ between the chain and the field:$\Gamma_{magn}=\Gamma_{o}\frac{\sin\left(2\varphi \right)}{2}$. *Γ*_*o*_ is calculated by summing the torques exerted between pairs of endosomes in the chain, each due to the magnetic dipole interaction with its neighbors:$\Gamma_{o}=\frac{3\mu_{o}m^{2}}{4\pi }\frac{N^{2}}{d^{3}}$, where *N* is the number of endosomes in the chain. When the field is continuously rotating, at a constant frequency*F*=*dθ*/*dt*, this magnetic moment equilibrates with the viscous torque:$\Gamma_{viscoel}=\kappa \eta V\frac{d\theta }{dt}$, where η is the viscosity of the medium surrounding the chain at the frequency of field rotation (0.2 Hz), V the volume of the chain, and κ a previously calibrated geometric factor (see 35,36 for more details). The measurement of the angular delay φ thus provides direct access to the viscous coefficient*η*: $\eta =\frac{\mu_{o}m^{2}}{32\pi \kappa }\frac{\sin\left(2\varphi \right)}{F}$(defined as equation 2). Such a measurement of the local viscosity was validated using magnetic beads (MyOne,*m*=10^−14^A m^2^), and performing the measure in glycerol (99.9% purity, *η*=0.35 Pa.sat 37°C). The magnetic local measurement performed retrieved a value*η*_*meas*_=0.359±0.05 Pa.s, in excellent agreement with the calibrated value.

### *In vitro* microtubules polymerization

The tubulin was provided from Cytoskeleton (Cytoskelton Inc., Denver USA). It was stored at the concentration of 10mg/ml at -80°C in BRB 1X buffer containing glycerol (50%) and guanosine triphosphate (GTP) (1mM). Microtubules were polymerized from a 1:4 mixture of rhodamine-labeled tubulin and unlabeled tubulin in BRB 1X buffer containing GTP (1mM), MgCl_2_ (25mM) and DMSO (1mM). This mixture was incubated at 37°C for 30min in order to allow for microtubule polymerization. The microtubules were then diluted at least 30 fold with a mixture of BRB 1X, Taxol (10µM) and dithiothreitol (DTT) (5mM) for stabilization. The microtubules were stored at room temperature and used within two weeks (adapted from [52]).

### *In vitro* Microtubule assay

Chambers used for the *in vitro* microtubule assay featured two overlaid silanized cover glasses (26*50 and 22*22mm). The inner space of chambers was created by positioning thin strips of parafilm between the cover glasses (Figure 6A). The chambers were sealed by heating at 100°C. The inner space between the cover glasses was less than 0.5mm corresponding to a volume of 10-15 µl. Different solutions were injected and penetrated into the inner space by capillarity.

First, we incubated the chamber with Anti-α-tubulin (5 min) followed by F127 (Pluronic) (10-15 min) to passivate the surface, and then microtubules (15 min), including a washing step with BRB 1X buffer between each incubation. After microtubule incubation, the chamber was washed with BRB 1X buffer containing Taxol (10µM) and DTT (5mM). The last step consisted in injecting the last solution and the photosensitizer (free in solution or inside endosomes) at different concentrations. Concerning the free photosensitizer condition, triton X-100 was added to the solution at 0.3% final concentration in order to prevent photosensitizer aggregation. For the endosome solution, the photosensitizer (TPCS2a) concentration was determined by fluorescence spectroscopy (SLM Aminco Bowan Series 2 spectrometer) at the excitation wavelength of 410 nm.

### Statistics

Data are presented as mean values ± standard error of the mean (SEM). Numbers of independent measurements were n > 30 for passive measurements, n > 25 for active measurements. t-test with Welch’s correction was performed to determine a significant difference between the test and control groups using Prism 3.0 version of GraphPad software (USA). A minimum of 99% confidence level was considered significant. *** indicatesp<0.001. ** indicatesp<0.01. * indicatesp<0.05. NS (Non Significant) indicatesp>0.05.
